# Supplementary material for: Effectiveness of the 2024–2025 KP.2 COVID-19 vaccines in the United States during long-term follow-up
Source: Nat Commun. 2025 Dec 24;17:1043. doi: 10.1038/s41467-025-67796-0 (PMC12847879; doi:10.1038/s41467-025-67796-0)
Supplement: Supplementary file 1 — Supplementary Information [file 41467_2025_67796_MOESM1_ESM.pdf]

Supplemental material for manuscript:

"Effectiveness of the 2024-2025 KP.2 COVID-19 Vaccines in the United States During Long-Term Follow-Up ".

Contents

Supplemental Table 1. Specification and emulation of a target randomized controlled trial of KP.2 COVID-19 vaccine versus no KP.2 COVID-19 vaccine with enrollment from 8/23/24 until 01/17/25 and follow-up extending to April 12, 2025: execution of 8 sequential trials 3

Supplemental Table 2. Baseline characteristics of match-eligible persons before matching, presented as person-trials 7

Supplemental Figure 1. Cumulative distribution function of the propensity scores in the Raw (match eligible) and Matched populations 10

Supplemental Figure 2. Love-Love plot comparing baseline covariates in the Raw (match eligible) and Matched populations. 11

Supplemental Table 3. Risk differences between the vaccinated and unvaccinated arms of the target trial emulation study in SARS-CoV-2 infection, SARS-CoV-2 associated ED/UC visit, SARS-CoV-2-associated hospitalization and SARS-CoV-2-associated deaths, and numbers needed to vaccinate to prevent an adverse outcome, extending to the end of the follow-up period through 4/12/2025 12

Supplemental Table 4. Estimated effectiveness of 2024-2025 COVID-19 vaccines, targeting the KP.2 variant of Omicron extending to the end of the follow-up period through 4/12/2025: subgroup results by age group, Care Assessment Need (CAN) score, time since prior vaccination and time since prior documented positive tests 13

Supplemental Figure 3. Estimated effectiveness (per-protocol analysis) of the 2024-2025 COVID-19 KP.2 vaccines against documented SARS-CoV-2 infection, SARS-CoV-2 associated ED/UC visits, SARS-CoV-2-associated hospitalization and SARS-CoV-2-associated death, during a study period extending from 08/23/2024 to 04/12/2025: identified all ED/UC visits and hospitalizations from -1 to +10 days of a positive test (rather than those that also had a documented COVID-19 code) 17

Supplemental Figure 4. Estimated effectiveness (per-protocol analysis) of the 2024-2025 COVID-19 KP.2 vaccines against documented SARS-CoV-2 infection, SARS-CoV-2 associated ED/UC visits, SARS-CoV-2-associated hospitalization and SARS-CoV-2-associated death, during a study period extending from 08/23/2024 to 04/12/2025: identified ED/UC visits and hospitalizations from -1 to +10 days of a positive test that had a documented acute respiratory infection code (rather than those that also had a documented COVID-19 code) 18

Supplemental Figure 5. Estimated effectiveness (per-protocol analysis) of the 2024-2025 COVID-19 KP.2 vaccines against documented SARS-CoV-2 infection, SARS-CoV-2 associated ED/UC visits, SARS-CoV-2-associated hospitalization and SARS-CoV-2-associated death, during a study period extending from 08/23/2024 to 04/12/2025: identified ED/UC visits and hospitalizations within ±1 day of a positive test that had a documented COVID-19 code (rather than those from -1 to +10 days from the positive test) 19

Supplemental Table 5. Comparison of COVID-19 VE for "early trials" with enrollment from 8/23/24 to 10/27/24 versus "late trials" with enrollment from 10/28/24 to 01/17/25 20

Supplemental Table 6. Differences between participants in early (from 8/23/24 to 10/27/24) versus late (from 10/28/24 to 01/17/25) trials 22

Supplemental Figure 6. Estimated effectiveness of the 2024-2025 COVID-19 KP.2 vaccines against documented SARS-CoV-2 infection, SARS-CoV-2 associated ED/UC visits, SARS-CoV-2-associated hospitalization and SARS-CoV-2-associated death, during a study period extending from 08/23/2024 to 04/12/2025: Per-protocol analysis performed by censoring the matched pair when the unvaccinated comparator crossed over to the vaccinated arm. 23

Supplemental Figure 7. Intention-to-treat analysis (participants analyzed in their assigned arms irrespective of crossover from unvaccinated to vaccinated arm) of estimated effectiveness of the 2024-2025 COVID-19 KP.2 vaccines against documented SARS-CoV-2 infection, SARS-CoV-2 associated ED/UC visits, SARS-CoV-2-associated hospitalization and SARS-CoV-2-associated death, during a study period extending from 08/23/2024 to 04/12/2025 24

Supplemental Table 6. COVID-19 diagnosis codes (ICD-10) 25

Supplemental Table 7. Acute respiratory infection diagnosis codes (ICD-10) – from Appaneal et al[1]............................ 25

Supplemental Table 1. Specification and emulation of a target randomized controlled trial of KP.2 COVID-19 vaccine versus no KP.2 COVID-19 vaccine with enrollment from 8/23/24 until 01/17/25 and follow-up extending to April 12, 2025: execution of 8 sequential trials

Target Trial Specification	Target Trial Emulation
**Eight sequential trials were executed each with a ~2-week* enrollment period as follows:**<br>**Trial 1: 08/23/24 to 09/29/24**<br>**Trial 2: 09/30/24 to 10/13/24**<br>**Trial 3: 10/14/24 to 10/27/24**<br>**Trial 4: 10/28/24 to 11/10/24**<br>**Trial 5: 11/11/24 to 11/24/24**<br>**Trial 6: 11/25/24 to 12/08/24**<br>**Trial 7: 12/09/24 to 12/22/24**<br>**Trial 8: 12/23/24 to 01/17/25**<br><br>**All eligibility & matching criteria were determined separately for each trial as of the first day of each trial (highlighted in red) and then eligibility reconfirmed as of the index date.**<br>**** The first and last trials extended longer than 2 weeks due to low number of vaccinations right after KP.2 vaccine introduction and decline in vaccination rate after December***	
**ELIGIBILITY CRITERIA**	
VA enrollees aged ≥18 years	Same
Assigned to VA primary care team in the prior 12 months AND has at least one VA primary care visit in the last 12 months	Same
At least one blood pressure measurement in the last 12 months AND weight in the last 5 years AND height ever documented	Same
Known residential address and VISN assignment	Same
Received at least one documented COVID-19 vaccine in the VA healthcare system at any time in the past	Same
Did not receive any COVID-19 vaccination in the prior 3 months	Same
Did not receive a Novavax JN.1 COVID-19 vaccine (2024-25 formulation) at any time before	
Did not receive a KP.2 COVID-19 vaccine at any time before	Same
Did not test positive for SARS-CoV-2 in the prior 3 months	Same
No inpatient Hospitalization in the prior 30 days	Same
**TREATMENT STRATEGIES**	
Received KP.2 COVID-19 vaccination (Pfizer-BioNTech or Moderna) from 08/23/24 until 01/17/25 versus no KP.2 COVID-19 vaccine as of their assigned index date	Same.
**TREATMENT ASSIGNMENT**	
**Randomization**<br>Eligible participants were randomly assigned to receive the KP.2 COVID-19 vaccine versus no KP.2 COVID-19 vaccine	**Eight sequential trials were executed each one (except the first and last) consisting of a ~2-week enrollment period beginning on 08/23/24 and continuing until 01/17/25. For each 2-week period, a two-step process was used to match each eligible KP.2 vaccine recipient to an eligible person who did not receive the KP.2 vaccine as of that period, in order to emulate the balance in baseline characteristics**

	**achieved by randomization, using exact matching followed by propensity score matching.**<br><br>*Exact-matching*<br>Within each 2-week trial, we first exact-matched each eligible participant who received KP.2 COVID-19 vaccine to all eligible participants who did not receive KP.2 COVID-19 vaccine using six factors:<br>1. Age Category (18-64, 65-74, ≥75)<br>2. **CAN score 3.0 (90-DAY mortality)** category (0-50, 51-89, ≥90)<br>3. VA Integrated Service Network (VISN), the 18 administrative regions of the VA<br>4. Time since most recent COVID-19 vaccine (90-182, 183-364 and ≥365 days prior to the beginning of each 2-week trial)<br>5. Timing of most recent SARS-CoV-2 positive test (90-182, 183-364 and ≥365 days, or no infection documented prior to the beginning of each 2-week trial)<br>6. Number of primary and specialty outpatient healthcare visits (excluding mental health) in the previous 1 year (0-4, 5-8, 9-15, ≥16)<br><br>*Propensity-score matching*<br>After exact-matching by these six factors, we performed an additional propensity score matching step ultimately aiming to identify the best-matching comparator. We used matching with replacement in a 1:1 variable ratio with a comparator randomly selected in the case of ties.<br>The characteristics included in the propensity score logistic regression model are:<br>1. Demographic:<br>a. age (continuous),<br>b. sex,<br>c. race<br>d. ethnicity<br>2. Urban versus rural residence based on RUCA codes<br>3. Distance to nearest VHA medical facility: <10, 10-24, 25-49, ≥50 miles<br>4. Substance use (documented in the prior 2 years)<br>a. tobacco,<br>b. alcohol use disorder,<br>c. other substance use disorder<br>5. Underlying CDC high-risk conditions (documented in the prior 2 years)<br>a. Body mass index: <18.5, 18.5-24.9, 25-29.9, 30-34.9, 35-39.9, ≥40 kg/m^2<br>b. Chronic kidney disease<br>c. Diabetes<br>d. Coronary heart disease

<table>
<tr><td></td><td>e. Congestive heart failure
f. Chronic lung disease
g. Dementia
6. Charlson Comorbidity Index (continuous)
7. CAN score (continuous)
8. Healthcare utilization: number of primary care encounters in the previous one year, categorized
9. Healthcare utilization: number of specialty care encounters other than mental health in the previous one year, categorized
10. Healthcare utilization: Number of hospitalizations in the last 12 months
11. Receipt of immunosuppressive or cancer medications in the prior 1 year
12. Number of prior COVID-19 vaccinations</td></tr>
<tr><th colspan="2">OUTCOMES</th></tr>
<tr><td><u>Primary Outcomes</u>:
1. SARS-CoV-2 infection, defined as the earliest positive, laboratory-confirmed SARS-CoV-2 test (nucleic acid amplification or antigen test) in a respiratory specimen beginning 10 days after the randomization date through the end of the follow-up period.
2. SARS-CoV-2 associated urgent care or emergency department (ED/UC) visit, defined as visit for COVID-19 within one day before or one day after the eligible positive SARS-CoV-2 test.
3. SARS-CoV-2 associated hospitalization, defined as hospitalization for COVID-19 within one day before or one day after the eligible positive SARS-CoV-2 test.
4. SARS-CoV-2 associated death, defined as death from any cause within 30 days after the eligible positive SARS-CoV-2 test.</td><td><u>Primary Outcomes</u>:
Same, except that outcomes are captured relative to the index date rather than the randomization date.</td></tr>
<tr><th colspan="2">FOLLOW-UP</th></tr>
<tr><td>Analysis limited to participants who remain uninfected by day 10 after randomization, since there is no expectation of protective effect within the first 10 days after vaccination.
Follow-up for outcomes starts on day 10 after randomization and extends through 04/12/2025</td><td>Same
An index date was assigned to unvaccinated participants which was the date of KP.2 vaccination of the person they we matched to. Matched unvaccinated and vaccinated participants had to continue to fulfill enrollment criteria as of that index date (i.e. remained alive, uninfected and unvaccinated since the beginning of the 2-week trial period)
Analysis was limited to matched pairs in which both persons remain uninfected by day 10 after the index date.
Follow-up for outcomes starts on day 10 after randomization and extends through 04/12/2025.</td></tr>
<tr><th colspan="2">CAUSAL CONTRASTS</th></tr>
<tr><td><u>Intention-to-treat (ITT) analysis</u>. Participants analyzed in the arm they were assigned at randomization, irrespective</td><td>We performed a per-protocol analysis as the primary analysis, because in the real-world there is no expectation for unvaccinated participants to remain unvaccinated and</td></tr>
</table>

of whether they continued to adhere to their assigned treatment.<br><br><u>Per-protocol analysis.</u> Participants censored when they deviate from their assigned strategy and inverse probability censoring weights applied to adjust for baseline and time-varying covariates associated with adherence.	we expected a high rate of cross over from the unvaccinated to the vaccinated arm. In the primary per-protocol analysis the matched unvaccinated participants were censored at the time of vaccination with application of inverse probability of censoring weights on a bi-weekly basis. We used the same baseline and time-varying covariates included in the matching process as well as calendar time since the start of the study on 23 August 2024, and calendar time squared, updated bi-weekly, with weights estimated using pooled logistic regression for matched unvaccinated comparators, while matched vaccinated persons had a censoring weight of 1. We performed two additional sensitivity analyses: a per-protocol analysis where matched groups were censored at the time of crossover of the unvaccinated participant and an intention-to-treat analysis, in which matched unvaccinated participants who were later vaccinated after the index date remained analyzed in the unvaccinated group
**STATISTICAL ANALYSIS**	
Vaccine effectiveness was estimated as 100x(1- risk ratio)	Same<br>Vaccine effectiveness was defined as 100 x (1 - risk ratio) where the risk ratio was calculated by dividing Aalen-Johansen estimates of period-specific cumulative incidences.

Supplemental Table 2. Baseline characteristics of match-eligible persons before matching, presented as person-trials

	KP.2 COVID-19 vaccination (person-trials=541213)	No KP.2 COVID-19 vaccination (person-trials=12735883)	Overall (person-trials=13277096)
**Mean age (SD), yr**	70.7 (11.6)	65.6 (14.6)	65.8 (14.5)
**Age group, no. (%)**			
18-64 yr	132546 (24.5%)	5234918 (41.1%)	5367464 (40.4%)
65-74 yr	166041 (30.7%)	3243359 (25.5%)	3409400 (25.7%)
≥ 75 yr	242626 (44.8%)	4257606 (33.4%)	4500232 (33.9%)
**Sex, no. (%)**			
Female	50399 (9.3%)	1348457 (10.6%)	1398856 (10.5%)
Male	490814 (90.7%)	11387426 (89.4%)	11878240 (89.5%)
**Race, no. (%)**			
American Indian, Alaska Native	3724 (0.7%)	87622 (0.7%)	91346 (0.7%)
Asian	7429 (1.4%)	181099 (1.4%)	188528 (1.4%)
Black	125968 (23.3%)	2796970 (22.0%)	2922938 (22.0%)
Native Hawaiian, Other Pacific Islander	4778 (0.9%)	123657 (1.0%)	128435 (1.0%)
White	358274 (66.2%)	8491387 (66.7%)	8849661 (66.7%)
Multiple	4574 (0.8%)	121440 (1.0%)	126014 (0.9%)
Missing/Unknown/Declined**	36466 (6.7%)	933708 (7.3%)	970174 (7.3%)
**Ethnicity, no. (%)**			
Hispanic/Latino	33987 (6.3%)	1075439 (8.4%)	1109426 (8.4%)
Not Hispanic/Latino	482884 (89.2%)	11049719 (86.8%)	11532603 (86.9%)
Missing/Unknown/Declined**	24342 (4.5%)	610725 (4.8%)	635067 (4.8%)
**Rurality, no. (%)**			
Urban	385082 (71.2%)	8895430 (69.8%)	9280512 (69.9%)
Rural	156131 (28.8%)	3840453 (30.2%)	3996584 (30.1%)
**US geographical region, no. (%)**			
West	125068 (23.1%)	2607148 (20.5%)	2732216 (20.6%)
Midwest	139377 (25.8%)	2528189 (19.9%)	2667566 (20.1%)
Northeast	99927 (18.5%)	2069368 (16.2%)	2169295 (16.3%)
South	176841 (32.7%)	5531178 (43.4%)	5708019 (43.0%)
**Drive distance to nearest VHA medical facility in miles, no. (%)**			
0-9	255098 (47.1%)	5657902 (44.4%)	5913000 (44.5%)
10-24	199250 (36.8%)	4936548 (38.8%)	5135798 (38.7%)
25-49	71604 (13.2%)	1782940 (14.0%)	1854544 (14.0%)
≥50	15261 (2.8%)	358493 (2.8%)	373754 (2.8%)
**Drive time to nearest VHA medical facility in minutes, no. (%)**			

0-9	122735 (22.7%)	2702948 (21.2%)	2825683 (21.3%)
10-19	226573 (41.9%)	5207804 (40.9%)	5434377 (40.9%)
20-59	180609 (33.4%)	4559213 (35.8%)	4739822 (35.7%)
≥60	11296 (2.1%)	265918 (2.1%)	277214 (2.1%)
**Smoking, no. (%)**			
Current Smoker	65328 (12.1%)	1919978 (15.1%)	1985306 (15.0%)
Former Smoker	257172 (47.5%)	5435100 (42.7%)	5692272 (42.9%)
Never Smoker	214193 (39.6%)	5230133 (41.1%)	5444326 (41.0%)
Unknown	4520 (0.8%)	150672 (1.2%)	155192 (1.2%)
**Alcohol Use Disorder†, no. (%)**	106441 (19.7%)	2791261 (21.9%)	2897702 (21.8%)
**Substance Use Disorder†, no. (%)**	72720 (13.4%)	1973523 (15.5%)	2046243 (15.4%)
**Body mass index, no. (%)**			
<18·5 kg/m^2	4953 (0.9%)	131275 (1.0%)	136228 (1.0%)
18·5-24·9 kg/m^2	103593 (19.1%)	2387245 (18.7%)	2490838 (18.8%)
25-29·9 kg/m^2	196932 (36.4%)	4527376 (35.5%)	4724308 (35.6%)
30-34·9 kg/m^2	142565 (26.3%)	3413299 (26.8%)	3555864 (26.8%)
35-39·9 kg/m^2	62312 (11.5%)	1510678 (11.9%)	1572990 (11.8%)
≥40 kg/m^2	30858 (5.7%)	766010 (6.0%)	796868 (6.0%)
**Chronic Kidney Disease†, no. (%)**	163194 (30.2%)	3183554 (25%)	3346748 (25.2%)
**Diabetes†, no. (%)**	228431 (42.2%)	4421458 (34.7%)	4649889 (35%)
**Coronary Heart Disease†, no. (%)**	203913 (37.7%)	3951816 (31%)	4155729 (31.3%)
**Congestive Heart Failure†, no. (%)**	69148 (12.8%)	1253026 (9.8%)	1322174 (10%)
**Chronic Lung Disease†, no. (%)**	137477 (25.4%)	2763046 (21.7%)	2900523 (21.8%)
**Dementia†, no. (%)**	17481 (3.2%)	343388 (2.7%)	360869 (2.7%)
**Received immunosuppressive or cancer medications in the last year, no. (%)**	57696 (10.7%)	1063464 (8.4%)	1121160 (8.4%)
**Charlson Comorbidity Index, mean (SD)**	2.5 (2.4)	2 (2.2)	2 (2.2)
**Care Assessment Need (CAN) score for 90-day mortality, no. (%)**			
0-50	182367 (33.7%)	6064850 (47.6%)	6247217 (47.1%)
51-89	284306 (52.5%)	5269358 (41.4%)	5553664 (41.8%)
90-99	74540 (13.8%)	1401675 (11.0%)	1476215 (11.1%)
**Primary care encounters in the last year, no. (%)**			
0-1	38183 (7.1%)	1483751 (11.7%)	1521934 (11.5%)
2-3	121499 (22.4%)	3403095 (26.7%)	3524594 (26.5%)
4-7	177639 (32.8%)	3960985 (31.1%)	4138624 (31.2%)
≥8	203892 (37.7%)	3888052 (30.5%)	4091944 (30.8%)

**Specialty care encounters in the last year, no. (%)**			
0-1	18469 (3.4%)	913352 (7.2%)	931821 (7.0%)
2-3	59820 (11.1%)	2193368 (17.2%)	2253188 (17.0%)
4-7	130570 (24.1%)	3510554 (27.6%)	3641124 (27.4%)
≥8	332354 (61.4%)	6118609 (48.0%)	6450963 (48.6%)
**Hospitalization in the last year, no. (%)**			
0	466184 (86.1%)	11228479 (88.2%)	11694663 (88.1%)
1	53449 (9.9%)	1074561 (8.4%)	1128010 (8.5%)
2-4	20242 (3.7%)	403495 (3.2%)	423737 (3.2%)
≥5	1338 (0.2%)	29348 (0.2%)	30686 (0.2%)
**Trial number and enrollment period, no. (%)**			
1: 08/23/24 to 09/29/24	110033 (20.3%)	1840168 (14.4%)	1950201 (14.7%)
2: 09/30/24 to 10/13/24	123195 (22.8%)	1750160 (13.7%)	1873355 (14.1%)
3: 10/14/24 to 10/27/24	97361 (18.0%)	1647411 (12.9%)	1744772 (13.1%)
4: 10/28/24 to 11/10/24	77452 (14.3%)	1583757 (12.4%)	1661209 (12.5%)
5: 11/11/24 to 11/24/24	54188 (10.0%)	1523683 (12.0%)	1577871 (11.9%)
6: 11/25/24 to 12/08/24	34283 (6.3%)	1497247 (11.8%)	1531530 (11.5%)
7: 12/08/24 to 12/22/24	32075 (5.9%)	1450875 (11.4%)	1482950 (11.2%)
8: 12/23/24 to 1/17/25	12626 (2.3%)	1442582 (11.3%)	1455208 (11.0%)
**Time since most recent prior COVID-19 vaccine, no. (%)**			
90-182 days	40435 (7.5%)	453738 (3.6%)	494173 (3.7%)
183-364 days	308163 (56.9%)	2666018 (20.9%)	2974181 (22.4%)
≥365 days	192615 (35.6%)	9616127 (75.5%)	9808742 (73.9%)
**Number of prior COVID-19 vaccination(s) no. (%)**			
1-2	15532 (2.9%)	3367283 (26.4%)	3382815 (25.5%)
3	43970 (8.1%)	3439080 (27.0%)	3483050 (26.2%)
≥4	481711 (89.0%)	5929520 (46.6%)	6411231 (48.3%)
**Time since most recent prior positive SARS-CoV-2 test, no. (%)**			
90-182 days	3662 (0.7%)	66179 (0.5%)	69841 (0.5%)
183-364 days	14447 (2.7%)	249797 (2.0%)	264244 (2.0%)
≥365 days	114983 (21.2%)	2692934 (21.1%)	2807917 (21.1%)
No prior documented infection	408121 (75.4%)	9726973 (76.4%)	10135094 (76.3%)

Supplemental Figure 1. Cumulative distribution function of the propensity scores in the Raw (match eligible) and Matched populations

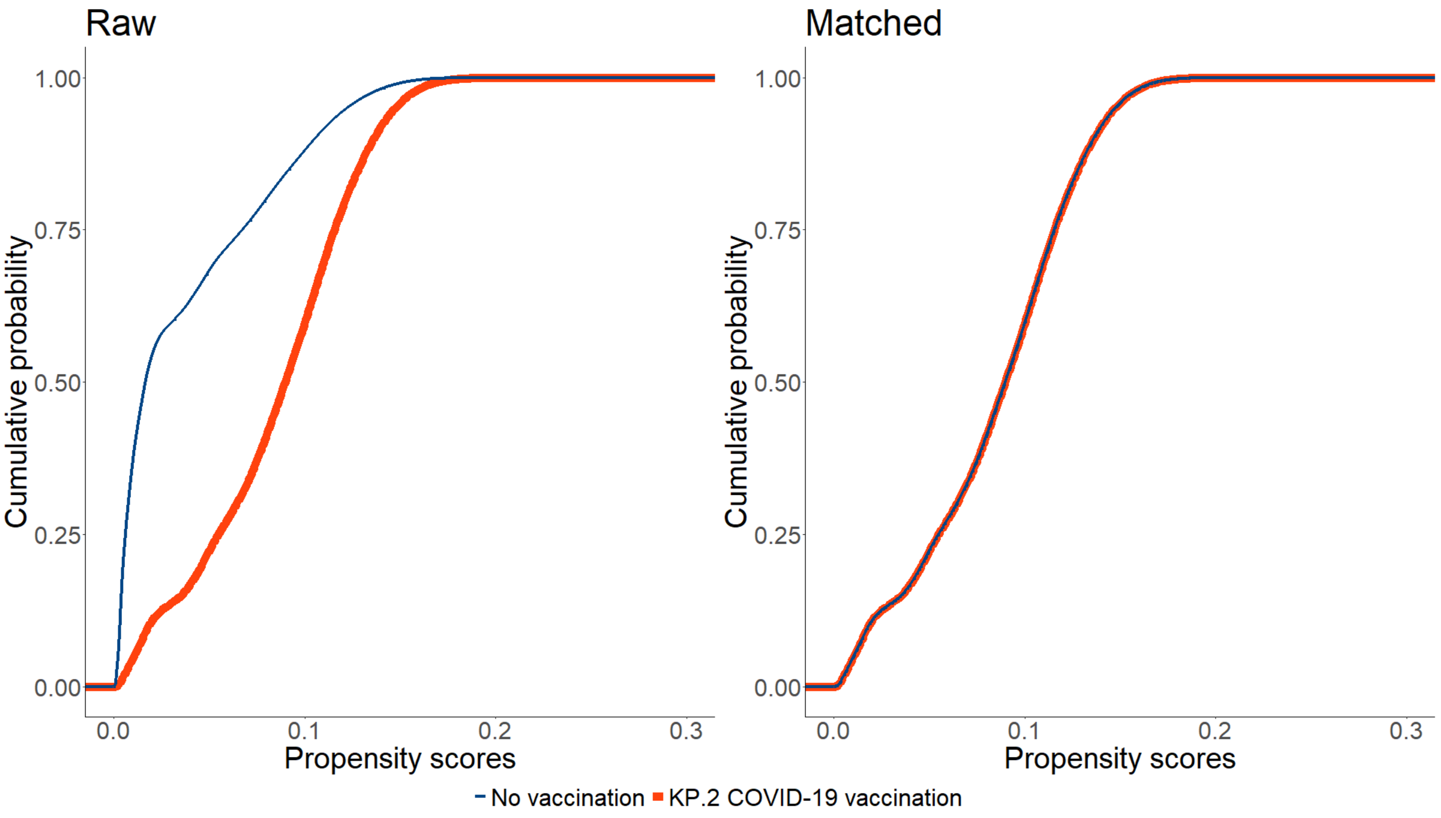

Supplemental Figure 2. Love-Love plot comparing baseline covariates in the Raw (match eligible) and Matched populations.

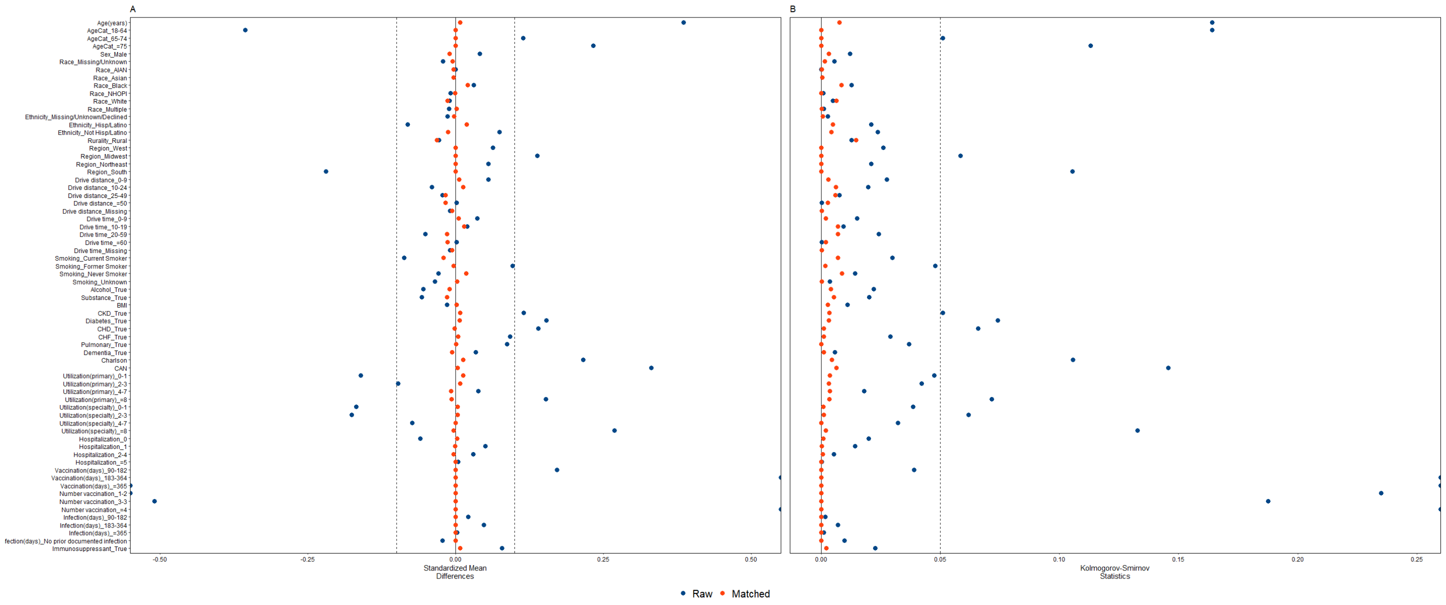

Supplemental Table 3. Risk differences between the vaccinated and unvaccinated arms of the target trial emulation study in SARS-CoV-2 infection, SARS-CoV-2 associated ED/UC visit, SARS-CoV-2-associated hospitalization and SARS-CoV-2-associated deaths, and numbers needed to vaccinate to prevent an adverse outcome, extending to the end of the follow-up period through 4/12/2025

	**KP.2 COVID-19 vaccination**	**No KP.2 COVID-19 vaccination**		
	**Cumulative incidence at the end of follow-up per 1000 persons (95% CI)**	**Cumulative incidence at the end of follow-up per 1000 persons (95% CI)**	**Risk difference (events/1000 person-years) (95% CI)**	**Number needed to vaccinate to prevent one outcome**
**SARS-CoV-2 infection**	7.74 (7.37 to 7.97)	9.29 (8.64 to 9.67)	-1.54 (-2.10 to -1.07)	649 (476 to 935)
**SARS-CoV-2-associated ED/UC visit**	4.24 (3.98 to 4.43)	5.36 (4.89 to 5.72)	-1.13 (-1.54 to -0.72)	885 (649 to 1389)
**SARS-CoV-2-associated hospitalization**	0.97 (0.88 to 1.07)	1.21 (1.04 to 1.38)	-0.24 (-0.40 to -0.07)	4,167 (2500 to 14,285)
**SARS-CoV-2-associated deaths**	0.11 (0.08 to 0.14)	0.31 (0.14 to 0.64)	-0.20 (-0.47 to -0.04)	5000 (2128 to 25,000)

Supplemental Table 4. Estimated effectiveness of 2024-2025 COVID-19 vaccines, targeting the KP.2 variant of Omicron extending to the end of the follow-up period through 4/12/2025: subgroup results by age group, Care Assessment Need (CAN) score, time since prior vaccination and time since prior documented positive tests

		KP.2 Vaccination		No KP.2 Vaccination		
**Outcome**	**Participants per arm, n**	**Events, n**	**Cumulative incidence at the end of follow-up per 1000 persons (95% CI)**	**Events, n**	**Cumulative incidence at the end of follow-up per 1000 persons (95% CI)**	**Vaccine Effectiveness (95% CI)**
**SARS-CoV-2 Infection**						
**Age group**						
18 to 64 years	131,779	696	6.31 (5.77 to 6.83)	889	8.09 (6.80 to 9.49)	21.99 (7.33 to 34.23)
65 to 74 years	164,828	1057	7.61 (7.03 to 8.10)	1358	9.45 (8.52 to 10.24)	19.45 (9.50 to 26.60)
≥75 years	240,859	1757	8.57 (7.99 to 8.92)	2106	9.91 (8.94 to 10.49)	13.50 (6.26 to 21.27)
**Care Assessment Need (CAN)**						
0-50	181,419	852	5.60 (5.18 to 6.02)	1083	7.15 (6.24 to 8.12)	21.65 (10.56 to 32.69)
51 to 89	282,932	1871	7.79 (7.32 to 8.16)	2295	9.13 (8.38 to 9.72)	14.66 (6.97 to 21.65)
90 to 99	73,115	787	12.91 (11.46 to 13.52)	975	15.52 (13.09 to 16.37)	16.87 (-382.70 to 26.12)
**Time since prior COVID-19 vaccine**						
90 to 365 days	345,835	2506	8.27 (7.84 to 8.54)	2656	9.11 (8.35 to 9.62)	9.17 (2.54 to 15.16)
>365 days	191,631	1004	6.72 (5.88 to 7.52)	1696	9.81 (8.60 to 10.87)	31.52 (20.21 to 41.97)
**Time since prior SARS-CoV-2 positive test**						
90 to 365 days	16,534	159	11.23 (9.37 to 13.02)	200	14.16 (10.89 to 17.56)	20.72 (-1.18 to 37.54)

>365 days	114,037	1068	11.10 (10.23 to 11.72)	1324	12.87 (11.55 to 13.81)	13.76 (4.07 to 22.50)
No infection	406,895	2283	6.66 (6.27 to 6.93)	2827	8.09 (7.40 to 8.59)	17.74 (10.65 to 24.31)
**SARS-CoV-2 Associated ED/UC visit**						
**Age group**						
18 to 64 years	131,779	352	3.34 (2.94 to 3.78)	469	4.63 (3.52 to 6.04)	27.79 (5.43 to 47.11)
65 to 74 years	164,828	589	4.26 (3.83 to 4.67)	777	5.48 (4.80 to 6.12)	22.26 (10.06 to 32.57)
≥75 years	240,859	944	4.69 (4.29 to 4.98)	1,221	5.78 (5.09 to 6.27)	18.85 (8.58 to 27.74)
**Care Assessment Need (CAN)**						
0-50	181,419	420	2.86 (2.56 to 3.19)	620	4.39 (3.60 to 5.32)	34.87 (19.57 to 47.26)
51 to 89	282,932	1,041	4.35 (4.02 to 4.64)	1,276	5.13 (4.59 to 5.62)	15.13 (5.65 to 23.36)
90 to 99	73,115	424	7.24 (6.18 to 7.90)	571	8.94 (7.37 to 9.72)	19.09 (5.88 to 32.44)
**Time since prior COVID-19 vaccine**						
90 to 365 days	345,835	1,308	4.42 (4.12 to 4.66)	1,502	5.20 (4.67 to 5.64)	15.03 (6.90 to 22.70)
>365 days	191,631	577	3.76 (3.26 to 4.27)	965	5.85 (4.80 to 6.93)	35.69 (19.92 to 49.17)
**Time since prior SARS-CoV-2 positive test**						
90 to 365 days	16,534	92	6.82 (5.38 to 8.38)	137	9.46 (6.94 to 12.34)	27.93 (1.35 to 47.87)
>365 days	114,037	617	6.59 (5.93 to 7.15)	787	7.81 (6.84 to 8.62)	15.64 (2.66 to 25.85)
No infection	406,895	1,176	3.46 (3.20 to 3.68)	1,543	4.52 (4.01 to 4.97)	23.43 (15.08 to 32.04)
**SARS-CoV-2 Associated hospitalization**						
**Age group**						
18 to 64 years	131,779	42	0.35 (0.25 to 0.47)	44	0.39 (0.23 to 0.67)	11.69 (-49.74 to 43.21)

65 to 74 years	164,828	134	0.89 (0.75 to 1.06)	158	1.09 (0.83 to 1.40)	17.75 (-8.76 to 39.06)
≥75 years	240,859	286	1.35 (1.18 to 1.52)	382	1.74 (1.43 to 2.05)	22.38 (5.47 to 34.37)
**Care Assessment Need (CAN)**						
0-50	181,419	40	0.26 (0.19 to 0.36)	31	0.21 (0.12 to 0.38)	-23.36 (-131.37 to 28.76)
51 to 89	282,932	218	0.85 (0.73 to 0.97)	274	1.06 (0.86 to 1.30)	20.30 (-0.21 to 34.50)
90 to 99	73,115	204	3.27 (2.72 to 3.70)	279	4.38 (3.38 to 5.11)	25.38 (6.55 to 38.65)
**Time since prior COVID-19 vaccine**						
90 to 365 days	345,835	314	1.01 (0.89 to 1.12)	357	1.20 (0.98 to 1.44)	16.31 (-2.77 to 29.15)
>365 days	191,631	148	0.88 (0.74 to 1.04)	227	1.23 (1.01 to 1.47)	28.49 (10.06 to 43.62)
**Time since prior SARS-CoV-2 positive test**						
90 to 365 days	16,534	19	1.28 (0.80 to 2.01)	36	2.47 (1.23 to 4.78)	47.96 (-2.31 to 73.34)
>365 days	114,037	143	1.39 (1.16 to 1.63)	173	1.61 (1.27 to 2.01)	14.00 (-10.95 to 32.71)
No infection	406,895	300	0.84 (0.74 to 0.95)	375	1.05 (0.86 to 1.25)	19.27 (3.19 to 33.63)
**SARS-CoV-2 Associated death**						
**Age group**						
18 to 74 years	296,607	12	0.05 (0.03 to 0.08)	**	0.05 (0.02 to 0.16)	11.77 (-314.81 to 71.14)
≥75 years	240,859	36	0.17 (0.12 to 0.24)	**	0.58 (0.27 to 1.19)	69.77 (29.00 to 85.72)
**Care Assessment Need (CAN)**						
0-50	181,419	**	**	**	**	**
51 to 89	282,932	**	0.07 (0.04 to 0.12)	**	0.23 (0.08 to 0.62)	68.95 (0.50 to 89.02)
90 to 99	73,115	**	0.49 (0.33 to 0.67)	**	0.80 (0.51 to 1.14)	39.00 (0.70 to 64.25)

**Time since prior COVID-19 vaccine**						
90 to 365 days	345,835	32	0.10 (0.07 to 0.14)	53	0.33 (0.13 to 0.79)	69.47 (18.94 to 86.03)
>365 days	191,631	16	0.12 (0.07 to 0.21)	39	0.19 (0.11 to 0.30)	33.84 (-27.79 to 69.19)
**Time since prior SARS-CoV-2 positive test**						
90 to 365 days	16,534	**	**	**	**	**
>365 days	114,037	**	0.14 (0.08 to 0.24)	**	0.21 (0.11 to 0.38)	33.51 (-55.44 to 71.34)
No infection	406,895	**	0.10 (0.07 to 0.14)	**	0.34 (0.13 to 0.83)	70.39 (23.59 to 87.23)

** Omitted to meet reporting requirements when cell counts are less than eleven

Supplemental Figure 3. Estimated effectiveness (per-protocol analysis) of the 2024-2025 COVID-19 KP.2 vaccines against documented SARS-CoV-2 infection, SARS-CoV-2 associated ED/UC visits, SARS-CoV-2-associated hospitalization and SARS-CoV-2-associated death, during a study period extending from 08/23/2024 to 04/12/2025: identified all ED/UC visits and hospitalizations from -1 to +10 days of a positive test (rather than those that also had a documented COVID-19 code)

		KP.2 COVID-19 vaccination		No KP.2 COVID-19 vaccination		
	N	No. of events	Cumulative incidence at end of follow-up per 1000 persons (95% CI)	No. of events	Cumulative incidence at end of follow-up per 1000 persons (95% CI)	Vaccine effectiveness (95% CI)
**SARS-CoV-2 infection**						
60-days of follow-up	537,466	1207	2.25 (2.12 to 2.38)	1829	3.28 (3.06 to 3.47)	31.28 (25.56 to 36.36)
90-days of follow-up	537,466	2013	3.76 (3.59 to 3.91)	2798	5.07 (4.76 to 5.32)	25.81 (20.90 to 29.75)
120-days of follow-up	537,466	2710	5.13 (4.91 to 5.29)	3582	6.61 (6.22 to 6.88)	22.44 (17.65 to 26.01)
Follow-up to end of study	537,466	3510	7.74 (7.37 to 7.97)	4353	9.29 (8.64 to 9.67)	16.60 (11.92 to 21.44)
**SARS-CoV-2 ED/UC visit**						
60-days of follow-up	537,466	738	1.38 (1.28 to 1.48)	1148	2.06 (1.90 to 2.22)	33.20 (25.91 to 39.58)
90-days of follow-up	537,466	1262	2.36 (2.22 to 2.48)	1785	3.24 (3.00 to 3.45)	27.20 (21.06 to 32.37)
120-days of follow-up	537,466	1694	3.20 (3.04 to 3.34)	2282	4.21 (3.92 to 4.45)	24.00 (18.23 to 28.99)
Follow-up to end of study	537,466	2225	5.03 (4.73 to 5.25)	2838	6.17 (5.66 to 6.54)	18.41 (12.00 to 24.29)
**SARS-CoV-2 hospitalization**						
60-days of follow-up	537,466	177	0.33 (0.28 to 0.38)	284	0.51 (0.43 to 0.59)	35.23 (22.16 to 47.58)
90-days of follow-up	537,466	300	0.56 (0.50 to 0.63)	424	0.77 (0.66 to 0.88)	27.12 (13.36 to 38.08)
120-days of follow-up	537,466	408	0.77 (0.70 to 0.84)	533	0.98 (0.86 to 1.11)	21.49 (9.31 to 32.30)
Follow-up to end of study	537,466	518	1.09 (0.98 to 1.18)	638	1.33 (1.14 to 1.51)	18.25 (5.24 to 28.21)
**SARS-CoV-2 deaths**						
60-days of follow-up	537,466	**	0.01 (0.01 to 0.03)	33	0.06 (0.04 to 0.09)	75.02 (48.01 to 91.63)
90-days of follow-up	537,466	15	0.03 (0.02 to 0.05)	53	0.10 (0.06 to 0.14)	71.02 (45.78 to 84.69)
120-days of follow-up	537,466	27	0.05 (0.03 to 0.07)	74	0.14 (0.10 to 0.20)	63.08 (43.73 to 77.89)
Follow-up to end of study	537,466	48	0.11 (0.08 to 0.14)	92	0.31 (0.14 to 0.64)	65.53 (27.79 to 83.37)

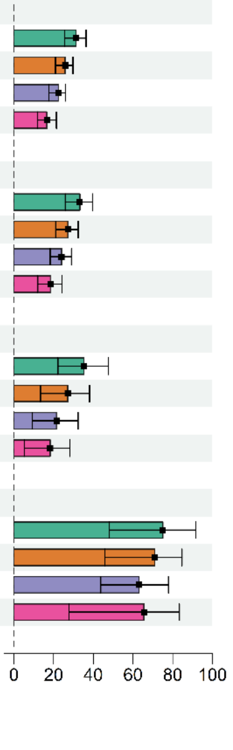

Supplemental Figure 4. Estimated effectiveness (per-protocol analysis) of the 2024-2025 COVID-19 KP.2 vaccines against documented SARS-CoV-2 infection, SARS-CoV-2 associated ED/UC visits, SARS-CoV-2-associated hospitalization and SARS-CoV-2-associated death, during a study period extending from 08/23/2024 to 04/12/2025: identified ED/UC visits and hospitalizations from -1 to +10 days of a positive test that had a documented acute respiratory infection code (rather than those that also had a documented COVID-19 code)

		KP.2 COVID-19 vaccination		No KP.2 COVID-19 vaccination		
	N	No. of events	Cumulative incidence at end of follow-up per 1000 persons (95% CI)	No. of events	Cumulative incidence at end of follow-up per 1000 persons (95% CI)	Vaccine effectiveness (95% CI)
**SARS-CoV-2 infection**						
60-days of follow-up	537,466	1207	2.25 (2.12 to 2.38)	1829	3.28 (3.06 to 3.47)	31.28 (25.56 to 36.36)
90-days of follow-up	537,466	2013	3.76 (3.59 to 3.91)	2798	5.07 (4.76 to 5.32)	25.81 (20.90 to 29.75)
120-days of follow-up	537,466	2710	5.13 (4.91 to 5.29)	3582	6.61 (6.22 to 6.88)	22.44 (17.65 to 26.01)
Follow-up to end of study	537,466	3510	7.74 (7.37 to 7.97)	4353	9.29 (8.64 to 9.67)	16.60 (11.92 to 21.44)
**SARS-CoV-2 ED/UC visit**						
60-days of follow-up	537,466	692	1.29 (1.20 to 1.39)	1093	1.96 (1.80 to 2.12)	34.22 (27.42 to 40.22)
90-days of follow-up	537,466	1189	2.22 (2.09 to 2.34)	1693	3.07 (2.84 to 3.28)	27.69 (21.75 to 33.19)
120-days of follow-up	537,466	1595	3.02 (2.85 to 3.15)	2161	3.99 (3.70 to 4.22)	24.45 (18.38 to 29.36)
Follow-up to end of study	537,466	2091	4.69 (4.42 to 4.89)	2687	5.85 (5.35 to 6.21)	19.77 (13.46 to 26.47)
**SARS-CoV-2 hospitalization**						
60-days of follow-up	537,466	172	0.32 (0.28 to 0.37)	277	0.50 (0.42 to 0.58)	35.44 (18.24 to 47.70)
90-days of follow-up	537,466	292	0.55 (0.48 to 0.61)	407	0.74 (0.63 to 0.85)	26.03 (12.22 to 36.92)
120-days of follow-up	537,466	396	0.75 (0.67 to 0.82)	508	0.93 (0.81 to 1.06)	20.00 (7.65 to 30.91)
Follow-up to end of study	537,466	497	1.04 (0.94 to 1.14)	606	1.25 (1.08 to 1.43)	16.81 (4.23 to 29.25)
**SARS-CoV-2 deaths**						
60-days of follow-up	537,466	**	0.01 (0.01 to 0.03)	33	0.06 (0.04 to 0.09)	75.02 (48.01 to 91.63)
90-days of follow-up	537,466	15	0.03 (0.02 to 0.05)	53	0.10 (0.06 to 0.14)	71.02 (45.78 to 84.69)
120-days of follow-up	537,466	27	0.05 (0.03 to 0.07)	74	0.14 (0.10 to 0.20)	63.08 (43.73 to 77.89)
Follow-up to end of study	537,466	48	0.11 (0.08 to 0.14)	92	0.31 (0.14 to 0.64)	65.53 (27.79 to 83.37)

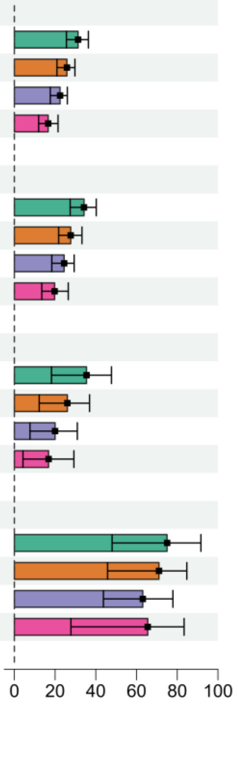

Supplemental Figure 5. Estimated effectiveness (per-protocol analysis) of the 2024-2025 COVID-19 KP.2 vaccines against documented SARS-CoV-2 infection, SARS-CoV-2 associated ED/UC visits, SARS-CoV-2-associated hospitalization and SARS-CoV-2-associated death, during a study period extending from 08/23/2024 to 04/12/2025: <u>identified ED/UC visits and hospitalizations within ±1 day of a positive test that had a documented COVID-19 code</u> (rather than those from -1 to +10 days from the positive test)

		KP.2 COVID-19 vaccination		No KP.2 COVID-19 vaccination		
	N	No. of events	Cumulative incidence at end of follow-up per 1000 persons (95% CI)	No. of events	Cumulative incidence at end of follow-up per 1000 persons (95% CI)	Vaccine effectiveness (95% CI)
**SARS-CoV-2 infection**						
60-days of follow-up	537,466	1207	2.25 (2.12 to 2.38)	1829	3.28 (3.06 to 3.47)	31.28 (25.56 to 36.36)
90-days of follow-up	537,466	2013	3.76 (3.59 to 3.91)	2798	5.07 (4.76 to 5.32)	25.81 (20.90 to 29.75)
120-days of follow-up	537,466	2710	5.13 (4.91 to 5.29)	3582	6.61 (6.22 to 6.88)	22.44 (17.65 to 26.01)
Follow-up to end of study	537,466	3510	7.74 (7.37 to 7.97)	4353	9.29 (8.64 to 9.67)	16.60 (11.92 to 21.44)
**SARS-CoV-2 ED/UC visit**						
60-days of follow-up	537,466	622	1.16 (1.07 to 1.25)	995	1.79 (1.63 to 1.93)	35.05 (27.72 to 41.54)
90-days of follow-up	537,466	1052	1.97 (1.84 to 2.08)	1529	2.77 (2.55 to 2.97)	29.15 (23.04 to 35.02)
120-days of follow-up	537,466	1411	2.67 (2.52 to 2.79)	1947	3.59 (3.32 to 3.81)	25.80 (19.65 to 30.87)
Follow-up to end of study	537,466	1841	4.13 (3.88 to 4.32)	2419	5.27 (4.80 to 5.63)	21.58 (14.45 to 27.57)
**SARS-CoV-2 hospitalization**						
60-days of follow-up	537,466	143	0.27 (0.23 to 0.31)	239	0.43 (0.36 to 0.51)	37.82 (23.12 to 49.83)
90-days of follow-up	537,466	247	0.46 (0.41 to 0.52)	336	0.61 (0.52 to 0.70)	23.92 (8.94 to 35.85)
120-days of follow-up	537,466	339	0.64 (0.57 to 0.71)	418	0.77 (0.66 to 0.88)	16.63 (2.04 to 29.28)
Follow-up to end of study	537,466	422	0.88 (0.79 to 0.97)	512	1.05 (0.89 to 1.21)	15.85 (2.78 to 26.98)
**SARS-CoV-2 deaths**						
60-days of follow-up	537,466	**	0.01 (0.01 to 0.03)	33	0.06 (0.04 to 0.09)	75.02 (48.01 to 91.63)
90-days of follow-up	537,466	15	0.03 (0.02 to 0.05)	53	0.10 (0.06 to 0.14)	71.02 (45.78 to 84.69)
120-days of follow-up	537,466	27	0.05 (0.03 to 0.07)	74	0.14 (0.10 to 0.20)	63.08 (43.73 to 77.89)
Follow-up to end of study	537,466	48	0.11 (0.08 to 0.14)	92	0.31 (0.14 to 0.64)	65.53 (27.79 to 83.37)

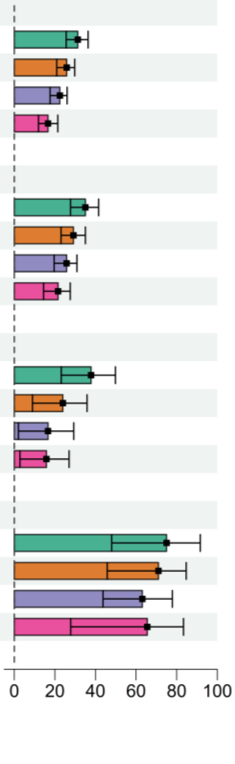

Supplemental Table 5. Comparison of COVID-19 VE for "early trials" with enrollment from 8/23/24 to 10/27/24 versus "late trials" with enrollment from 10/28/24 to 01/17/25

		KP.2 Vaccination		No KP.2 Vaccination		
**Outcome**	**Participants per arm, n**	**Events,n**	**Cumulative incidence at the end of follow-up per 1000 persons (95% CI)**	**Events, n**	**Cumulative incidence at the end of follow-up per 1000 persons (95% CI)**	**Vaccine Effectiveness (95% CI)**
**SARS-CoV-2 Infection**						
**Follow-up to 60-days**						
Early Trials	328,590	714	2.18 (2.02-2.34)	858	2.81 (2.57-3.06)	22.58 (14.03-30.08)
Late Trials	208,876	493	2.37 (2.16-2.58)	970	3.82 (3.49-4.12)	37.97 (29.94-45.01)
**Follow-up to 90-days**						
Early	328,590	1,284	3.93 (3.70-4.13)	1,447	4.87 (4.49-5.19)	19.35 (13.12-24.85)
Late	208,876	729	3.51 (3.25-3.75)	1,351	5.32 (4.91-5.68)	34.11 (27.45-39.71)
**SARS-CoV-2 Associated ED/UC visit**						
**Follow-up to 60-days**						
Early	328,590	361	1.10 (0.99-1.22)	462	1.52 (1.35-1.71)	27.76 (15.90-37.31)
Late	208,876	278	1.33 (1.18-1.50)	549	2.16 (1.92-2.40)	38.20 (27.89-47.32)
**Follow-up to 90-days**						
Early	328,590	664	2.03 (1.87-2.18)	795	2.68 (2.41-2.95)	24.39 (15.87-32.37)
Late	208,876	413	1.99 (1.80-2.18)	770	3.03 (2.73-3.32)	34.54 (25.56-42.84)
**SARS-CoV-2 Associated hospitalization**						
**Follow-up to 60-days**						
Early	328,590	93	0.28 (0.23-0.35)	128	0.42 (0.34-0.53)	33.00 (10.19-50.26)
Late	208,876	67	0.32 (0.25-0.41)	137	0.54 (0.43-0.67)	40.34 (18.65-55.84)
**Follow-up to 90-days**						
Early	328,590	166	0.51 (0.43-0.59)	208	0.70 (0.58-0.84)	27.82 (10.04-41.27)
Late	208,876	104	0.50 (0.41-0.60)	183	0.72 (0.58-0.88)	30.73 (10.98-47.56)

**SARS-CoV-2 Associated death**						
**Follow-up to 60-days**						
Early	328,590	**	0.01 (0.00-0.03)	19	0.07 (0.04-0.12)	86.08 (55.36-100.00)
Late	208,876	**	0.02 (0.01-0.06)	13	0.05 (0.03-0.10)	54.32 (-21.56-90.01)
**Follow-up to 90-days**						
Early	328,590	**	0.02 (0.01-0.04)	27	0.09 (0.05-0.15)	76.87 (51.53-93.58)
Late	208,876	**	0.04 (0.02-0.08)	25	0.10 (0.06-0.17)	61.90 (12.70-86.46)

Supplemental Table 6. Differences between participants in early (from 8/23/24 to 10/27/24) versus late (from 10/28/24 to 01/17/25) trials

	Early (N=657180)	Late (N=417752)	Overall (N=1074932)
**Received immunosuppressive or cancer medications in the last year, no. (%)**	34931 (5.3%)	20990 (5%)	55921 (5.2%)
**Age group (years), no. (%)**			
18-64	145576 (22.2%)	117982 (28.2%)	263558 (24.5%)
65-74	201244 (30.6%)	128412 (30.7%)	329656 (30.7%)
≥75	310360 (47.2%)	171358 (41.0%)	481718 (44.8%)
**Care Assessment Need (CAN) score for 90-day mortality, no. (%)**			
0-50	210302 (32.0%)	152536 (36.5%)	362838 (33.8%)
51-89	355336 (54.1%)	210528 (50.4%)	565864 (52.6%)
≥90	91542 (13.9%)	54688 (13.1%)	146230 (13.6%)
**Time since most recent prior COVID-19 vaccine in days, no. (%)**			
90-182	51786 (7.9%)	27078 (6.5%)	78864 (7.3%)
183-364	445700 (67.8%)	167106 (40.0%)	612806 (57.0%)
≥365	159694 (24.3%)	223568 (53.5%)	383262 (35.7%)

Supplemental Figure 6. Estimated effectiveness of the 2024-2025 COVID-19 KP.2 vaccines against documented SARS-CoV-2 infection, SARS-CoV-2 associated ED/UC visits, SARS-CoV-2-associated hospitalization and SARS-CoV-2-associated death, during a study period extending from 08/23/2024 to 04/12/2025: <u>Per-protocol analysis performed by censoring the matched pair</u> when the unvaccinated comparator crossed over to the vaccinated arm.

		KP.2 COVID-19 vaccination		No KP.2 COVID-19 vaccination		
	N	No. of events	Cumulative incidence at end of follow-up per 1000 persons (95% CI)	No. of events	Cumulative incidence at end of follow-up per 1000 persons (95% CI)	Vaccine effectiveness (95% CI)
**SARS-CoV-2 infection**						
60-days of follow-up	537,466	992	2.22 (2.08 to 2.36)	1438	3.21 (3.01 to 3.40)	30.98 (24.73 to 36.38)
90-days of follow-up	537,466	1529	3.61 (3.42 to 3.78)	2108	4.97 (4.67 to 5.19)	27.26 (22.16 to 31.95)
120-days of follow-up	537,466	1956	4.84 (4.60 to 5.03)	2622	6.46 (6.09 to 6.71)	25.15 (20.27 to 29.95)
Follow-up to end of study	537,466	2379	7.30 (6.82 to 7.64)	3117	9.12 (8.49 to 9.49)	19.98 (13.72 to 25.94)
**SARS-CoV-2 ED/UC visit**						
60-days of follow-up	537,466	538	1.20 (1.10 to 1.31)	790	1.78 (1.63 to 1.92)	32.25 (24.49 to 39.46)
90-days of follow-up	537,466	827	1.95 (1.82 to 2.08)	1170	2.77 (2.56 to 2.95)	29.48 (23.83 to 35.21)
120-days of follow-up	537,466	1049	2.59 (2.42 to 2.74)	1455	3.60 (3.34 to 3.81)	28.04 (21.57 to 33.61)
Follow-up to end of study	537,466	1281	3.99 (3.67 to 4.26)	1757	5.26 (4.80 to 5.61)	24.19 (16.28 to 31.13)
**SARS-CoV-2 hospitalization**						
60-days of follow-up	537,466	138	0.31 (0.26 to 0.36)	208	0.46 (0.40 to 0.54)	33.53 (17.07 to 45.68)
90-days of follow-up	537,466	209	0.49 (0.43 to 0.56)	294	0.69 (0.59 to 0.79)	28.39 (14.38 to 39.53)
120-days of follow-up	537,466	272	0.67 (0.59 to 0.75)	358	0.87 (0.76 to 0.99)	23.15 (8.68 to 34.25)
Follow-up to end of study	537,466	321	0.91 (0.79 to 1.03)	420	1.18 (1.02 to 1.34)	23.08 (7.54 to 35.41)
**SARS-CoV-2 deaths**						
60-days of follow-up	537,466	**	0.01 (0.01 to 0.03)	26	0.06 (0.04 to 0.09)	76.62 (47.01 to 92.22)
90-days of follow-up	537,466	12	0.03 (0.02 to 0.05)	39	0.09 (0.06 to 0.14)	68.41 (41.61 to 86.16)
120-days of follow-up	537,466	19	0.05 (0.03 to 0.08)	53	0.13 (0.09 to 0.19)	63.19 (40.05 to 80.25)
Follow-up to end of study	537,466	29	0.10 (0.06 to 0.14)	64	0.26 (0.14 to 0.48)	63.82 (29.70 to 81.93)

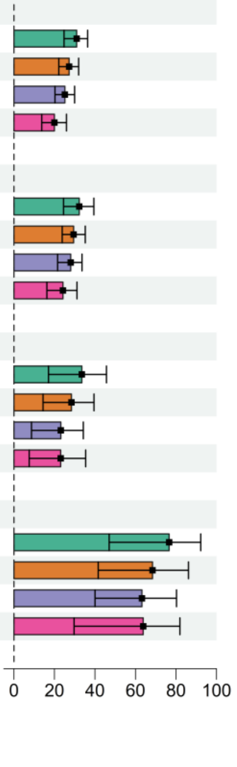

** Omitted to meet reporting requirements when cell counts are less than eleven

Supplemental Figure 7. Intention-to-treat analysis (participants analyzed in their assigned arms irrespective of crossover from unvaccinated to vaccinated arm) of estimated effectiveness of the 2024-2025 COVID-19 KP.2 vaccines against documented SARS-CoV-2 infection, SARS-CoV-2 associated ED/UC visits, SARS-CoV-2-associated hospitalization and SARS-CoV-2-associated death, during a study period extending from 08/23/2024 to 04/12/2025

		KP.2 COVID-19 vaccination		No KP.2 COVID-19 vaccination		
	N	No. of events	Cumulative incidence at end of follow-up per 1000 persons (95% CI)	No. of events	Cumulative incidence at end of follow-up per 1000 persons (95% CI)	Vaccine effectiveness (95% CI)
**SARS-CoV-2 infection**						
60-days of follow-up	537,466	1207	2.25 (2.12 to 2.38)	1657	3.10 (2.91 to 3.26)	27.30 (21.67 to 32.29)
90-days of follow-up	537,466	2013	3.76 (3.59 to 3.91)	2590	4.86 (4.59 to 5.05)	22.49 (17.28 to 27.27)
120-days of follow-up	537,466	2710	5.13 (4.91 to 5.29)	3341	6.34 (6.01 to 6.55)	19.07 (14.40 to 23.45)
Follow-up to end of study	537,466	3510	7.74 (7.37 to 7.97)	4140	9.03 (8.49 to 9.32)	14.20 (8.95 to 19.21)
**SARS-CoV-2 ED/UC visit**						
60-days of follow-up	537,466	639	1.19 (1.10 to 1.28)	912	1.70 (1.57 to 1.83)	30.07 (21.43 to 37.40)
90-days of follow-up	537,466	1077	2.01 (1.89 to 2.13)	1435	2.69 (2.50 to 2.85)	25.15 (19.07 to 31.13)
120-days of follow-up	537,466	1445	2.73 (2.58 to 2.86)	1846	3.50 (3.27 to 3.67)	21.87 (16.53 to 27.12)
Follow-up to end of study	537,466	1885	4.24 (3.98 to 4.43)	2323	5.07 (4.71 to 5.32)	16.47 (9.59 to 21.93)
**SARS-CoV-2 hospitalization**						
60-days of follow-up	537,466	160	0.30 (0.25 to 0.35)	241	0.45 (0.39 to 0.52)	33.72 (18.54 to 46.04)
90-days of follow-up	537,466	270	0.50 (0.45 to 0.57)	365	0.68 (0.60 to 0.77)	26.18 (13.23 to 36.89)
120-days of follow-up	537,466	368	0.69 (0.62 to 0.77)	460	0.87 (0.77 to 0.97)	20.04 (7.31 to 29.67)
Follow-up to end of study	537,466	462	0.97 (0.88 to 1.07)	561	1.15 (1.02 to 1.27)	15.44 (4.28 to 26.15)
**SARS-CoV-2 deaths**						
60-days of follow-up	537,466	**	0.01 (0.01 to 0.03)	28	0.05 (0.03 to 0.08)	71.48 (41.77 to 89.02)
90-days of follow-up	537,466	15	0.03 (0.02 to 0.05)	44	0.08 (0.06 to 0.12)	65.98 (40.68 to 83.38)
120-days of follow-up	537,466	27	0.05 (0.03 to 0.07)	61	0.12 (0.08 to 0.16)	55.77 (31.58 to 74.43)
Follow-up to end of study	537,466	48	0.11 (0.08 to 0.14)	77	0.20 (0.13 to 0.30)	46.20 (13.10 to 67.30)

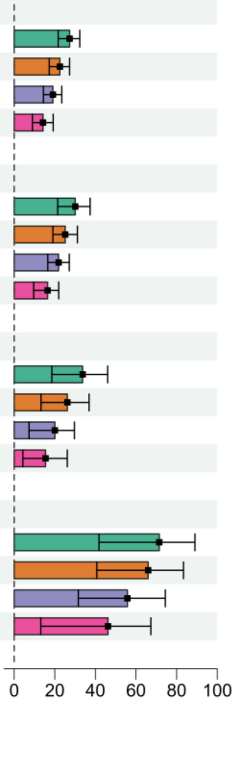

** Omitted to meet reporting requirements when cell counts are less than eleven

Supplemental Table 6. COVID-19 diagnosis codes (ICD-10)

ICD-10 code	Diagnosis
U07.1	COVID-19
U07.2	COVID-19, virus not identified (clinically diagnosed) |
J12.82	Pneumonia due to coronavirus disease 2019
M35.81	Multisystem inflammatory syndrome following COVID-19

Supplemental Table 7. Acute respiratory infection diagnosis codes (ICD-10) – from Appaneal et al[1].

ICD-10 code	Diagnosis
A22.1	Pulmonary anthrax
A37.00	Whooping cough due to Bordetella pertussis without pneumonia
A37.01	Whooping cough due to Bordetella pertussis with pneumonia
A37.10	Whooping cough due to Bordetella parapertussis without pneumonia
A37.11	Whooping cough due to Bordetella parapertussis with pneumonia
A37.80	Whooping cough due to other Bordetella species without pneumonia
A37.81	Whooping cough due to other Bordetella species with pneumonia
A37.90	Whooping cough, unspecified species without pneumonia
A37.91	Whooping cough, unspecified species with pneumonia
A48.1	Legionnaires' disease
B25.0	Cytomegaloviral pneumonitis
B34.2	Coronavirus infection, unspecified
B44.0	Invasive pulmonary aspergillosis
B77.81	Ascariasis pneumonia
B97.29	Other coronavirus as the cause of diseases classified elsewhere
J00*	Acute nasopharyngitis [common cold]
J01*	Acute sinusitis
J02*	Acute pharyngitis
J03*	Acute tonsillitis
J04*	Acute laryngitis and tracheitis
J05*	Acute obstructive laryngitis [croup] and epiglottitis
J06*	Acute upper respiratory infections of multiple and unspecified sites
J09.X1	Influenza due to novel influenza A virus with pneumonia
J09.X2	Influenza due to identified novel influenza A virus with other respiratory manifestations
J09.X3	Influenza due to identified novel influenza A virus with gastrointestinal manifestations
J09.X9	Influenza due to identified novel influenza A virus with other manifestations
J10.00	Influenza due to other identified influenza virus with pneumonia
J10.01	Influenza due to other identified influenza virus with the same other identified influenza virus pneumonia
J10.08	Influenza due to other identified influenza virus with other specified pneumonia
J10.1	Influenza due to other identified influenza virus with other respiratory manifestations

J10.2	Influenza due to other identified influenza virus with gastrointestinal manifestations
J10.8*	Influenza due to other identified influenza virus with other manifestations
J10.81	Influenza due to other identified influenza virus with encephalopathy
J10.82	Influenza due to other identified influenza virus with myocarditis
J10.83	Influenza due to other identified influenza virus with otitis media
J10.89	Influenza due to other identified influenza virus with other manifestations
J11.00	Influenza due to unidentified influenza virus with pneumonia
J11.08	Influenza due to unidentified influenza virus with specified pneumonia
J11.1	Influenza due to unidentified influenza virus with other respiratory manifestations
J11.2	Influenza due to unidentified influenza virus with gastrointestinal manifestations
J11.81	Influenza due to unidentified influenza virus with encephalopathy
J11.82	Influenza due to unidentified influenza virus with myocarditis
J11.83	Influenza due to unidentified influenza virus with otitis media
J11.89	Influenza due to unidentified influenza virus with other manifestations
J12.0	Adenoviral pneumonia
J12.1	Respiratory syncytial virus pneumonia
J12.2	Parainfluenza virus pneumonia
J12.3	Human metapneumovirus pneumonia
J12.81	Pneumonia due to SARS-associated coronavirus
J12.82	Pneumonia due to coronavirus disease 2019
J12.89	Other viral pneumonia
J12.9	Viral pneumonia, unspecified
J13	Pneumonia due to Streptococcus pneumoniae
J14	Pneumonia due to Hemophilus influenzae
J15.0	Pneumonia due to Klebsiella pneumoniae
J15.1	Pneumonia due to Pseudomonas
J15.20	Pneumonia due to Staphylococcus, unspecified
J15.211	Pneumonia due to methicillin susceptible Staphylococcus aureus
J15.212	Pneumonia due to methicillin resistant Staphylococcus aureus
J15.29	Pneumonia due to other Staphylococcus
J15.3	Pneumonia due to Streptococcus, group b
J15.4	Pneumonia due to other Streptococci
J15.5	Pneumonia due to Escherichia coli
J15.6	Pneumonia due to other aerobic gram-negative bacteria
J15.7	Pneumonia due to Mycoplasma pneumoniae
J15.8	Pneumonia due to other specified bacteria
J15.9	Unspecified bacterial pneumonia
J16.0	Chlamydial pneumonia
J16.8	Pneumonia due to other specified infectious organisms
J17	Pneumonia in diseases classified elsewhere
J18.0	Bronchopneumonia, unspecified organism
J18.1	Lobar pneumonia, unspecified organism
J18.2	Hypostatic pneumonia, unspecified organism

J18.8	Other pneumonia, unspecified organism
J18.9	Pneumonia, unspecified organism
J20.0	Acute bronchitis due to Mycoplasma pneumoniae
J20.1	Acute bronchitis due to Hemophilus influenzae
J20.2	Acute bronchitis due to Streptococcus
J20.3	Acute bronchitis due to coxsackievirus
J20.4	Acute bronchitis due to parainfluenza virus
J20.5	Acute bronchitis due to respiratory syncytial virus
J20.6	Acute bronchitis due to rhinovirus
J20.7	Acute bronchitis due to echovirus
J20.8	Acute bronchitis due to other specified organisms
J20.9	Acute bronchitis, unspecified
J21.*	Acute bronchiolitis
J21.0	Acute bronchiolitis due to respiratory syncytial virus
J21.1	Acute bronchiolitis due to human metapneumovirus
J21.8	Acute bronchiolitis due to other specified organisms
J21.9	Acute bronchiolitis, unspecified
J22	Unspecified acute lower respiratory infection
J80	Acute respiratory distress syndrome
J96.00	Acute respiratory failure unspecified whether with hypoxia or hypercapnia
J96.01	Acute respiratory failure with hypoxia
J96.02	Acute respiratory failure with hypercapnia
J96.10	Chronic respiratory failure, unspecified with hypoxia or hypercapnia
J96.11	Chronic respiratory failure with hypoxia
J96.12	Chronic respiratory failure with hypercapnia
J96.20	Acute and chronic respiratory failure, unspecified with hypoxia or hypercapnia
J96.21	Acute and chronic respiratory failure with hypoxia
J96.22	Acute and chronic respiratory failure with hypercapnia
J96.90	Respiratory failure, unspecified, unspecified with hypoxia or hypercapnia
J96.91	Respiratory failure, unspecified with hypoxia
J96.92	Respiratory failure, unspecified with hypercapnia
M35.81	Multisystem inflammatory syndrome following COVID-19
R04.2	Hemoptysis
R05	Cough
R05.1	Acute cough
R05.2	Subacute cough
R05.3	Chronic cough
R05.4	Cough syncope
R05.8	Other specified cough
R05.9	Cough, unspecified
R06.00	Dyspnea/abnormalities of breathing unspecified
R06.02	Shortness of breath
R06.03	Acute respiratory distress
R06.09	Other forms of dyspnea

R06.1	Stridor
R06.82	Tachypnea, not elsewhere classified
R06.89	Other abnormalities of breathing
R07.1	Chest pain on breathing
R09.0*	Asphyxia and hypoxemia
R09.01	Asphyxia
R09.02	Hypoxemia
R09.1	Pleurisy
R09.2	Respiratory arrest
R50.9	Fever, unspecified
U04*	SARS (WHO 2019)
U04.9	SARS, unspecified (WHO 2019)
U07.1	COVID-19
U07.2	COVID-19, virus not identified (clinically diagnosed) |

1. Appaneal HJ, Lopes VV, Puzniak L, et al. Early effectiveness of the BNT162b2 KP.2 vaccine against COVID-19 in the US Veterans Affairs Healthcare System. Nat Commun 2025;16:4033.
