# Supplementary material for: Effectiveness of the 2024–2025 KP.2 COVID-19 vaccines in the United States during long-term follow-up
Source: Nat Commun. 2025 Dec 24;17:1043. doi: 10.1038/s41467-025-67796-0 (PMC12847879; doi:10.1038/s41467-025-67796-0)
Supplement: Supplementary file 2 — Reporting Summary [file 41467_2025_67796_MOESM2_ESM.pdf]

nature portfolio

Corresponding author(s): George N. Ioannou

Last updated by author(s): Oct 21, 2025

Reporting Summary

Nature Portfolio wishes to improve the reproducibility of the work that we publish. This form provides structure for consistency and transparency in reporting. For further information on Nature Portfolio policies, see our Editorial Policies and the Editorial Policy Checklist.

Statistics

For all statistical analyses, confirm that the following items are present in the figure legend, table legend, main text, or Methods section.

n/a	Confirmed	
☐	☒	The exact sample size (*n*) for each experimental group/condition, given as a discrete number and unit of measurement
☐	☒	A statement on whether measurements were taken from distinct samples or whether the same sample was measured repeatedly
☒	☐	The statistical test(s) used AND whether they are one- or two-sided<br>*Only common tests should be described solely by name; describe more complex techniques in the Methods section.*
☐	☒	A description of all covariates tested
☐	☒	A description of any assumptions or corrections, such as tests of normality and adjustment for multiple comparisons
☐	☒	A full description of the statistical parameters including central tendency (e.g. means) or other basic estimates (e.g. regression coefficient) AND variation (e.g. standard deviation) or associated estimates of uncertainty (e.g. confidence intervals)
☒	☐	For null hypothesis testing, the test statistic (e.g. *F*, *t*, *r*) with confidence intervals, effect sizes, degrees of freedom and *P* value noted<br>*Give P values as exact values whenever suitable.*
☒	☐	For Bayesian analysis, information on the choice of priors and Markov chain Monte Carlo settings
☒	☐	For hierarchical and complex designs, identification of the appropriate level for tests and full reporting of outcomes
☒	☐	Estimates of effect sizes (e.g. Cohen's *d*, Pearson's *r*), indicating how they were calculated

Our web collection on statistics for biologists contains articles on many of the points above.

Software and code

Policy information about availability of computer code

Data collection	Data collection and analysis was performed using standard R software (version 4.4.1) and packages MatchIt (version 4.5.5), survival (version 3.7-0), boot (version 1.3-31), and speedglm (version 0.3-5). The code is available from the corresponding author upon request.
Data analysis	Data collection and analysis was performed using standard R software (version 4.4.1) and packages MatchIt (version 4.5.5), survival (version 3.7-0), boot (version 1.3-31), and speedglm (version 0.3-5). The code is available from the corresponding author upon request.

For manuscripts utilizing custom algorithms or software that are central to the research but not yet described in published literature, software must be made available to editors and reviewers. We strongly encourage code deposition in a community repository (e.g. GitHub). See the Nature Portfolio guidelines for submitting code & software for further information.

Data

Policy information about availability of data

All manuscripts must include a data availability statement. This statement should provide the following information, where applicable:

- Accession codes, unique identifiers, or web links for publicly available datasets
- A description of any restrictions on data availability
- For clinical datasets or third party data, please ensure that the statement adheres to our policy

The data supporting the findings of this study are not publicly available due to the inclusion of identifiable protected health information from the Veterans Health Administration. Privacy regulations prevent the open sharing of the individual-level data used in this study and any data covered under these regulations cannot be

shared. Other investigators can obtain access to the VHA data sources that we used, by directly applying through the Veterans Affairs Central Institutional Review Board

Research involving human participants, their data, or biological material

Policy information about studies with human participants or human data. See also policy information about sex, gender (identity/presentation), and sexual orientation and race, ethnicity and racism.

Reporting on sex and gender	Sex is documented in this study.<br>This is based on self-reported sex, as reported by Veterans who enrolled in VHA care.<br>Only Male and Female sex are being self-reported and available
Reporting on race, ethnicity, or other socially relevant groupings	Race and ethnicity are being documented in this study separately, as self reported by VHA enrollees.<br>We used all the available documented categories in VHA data<br>For Race, the categories are as follows:<br>American Indian, Alaska Native<br>Asian<br>Black<br>Native Hawaiian, Other Pacific Islander<br>White<br>Multiple<br>Declined/Unknown/Missing<br><br>For Ethnicity, the categories are as follows:<br>Hispanic/Latino<br>Not Hispanic/Latino<br>Declined/Unknown/Missing
Population characteristics	Matched participants were 90.9% male, 66.5% White, 22.8% Black, 6.0% Hispanic/Latino, and had a mean age of 70.7 years.
Recruitment	In this observational study, we identified all vaccine recipients and matched unvaccinated persons who fulfilled the eligibility criteria of the study, as outlined in Supplemental Table 1.
Ethics oversight	The protocol was approved by the Research and Development Committee and the Institutional Review Board of the Veterans Affairs Puget Sound Health Care System

Note that full information on the approval of the study protocol must also be provided in the manuscript.

Field-specific reporting

Please select the one below that is the best fit for your research. If you are not sure, read the appropriate sections before making your selection.

☒ Life sciences ☐ Behavioural & social sciences ☐ Ecological, evolutionary & environmental sciences

For a reference copy of the document with all sections, see nature.com/documents/nr-reporting-summary-flat.pdf

Life sciences study design

All studies must disclose on these points even when the disclosure is negative.

Sample size	All VHA enrollees who fulfilled the study's eligibility criteria as listed in Supplemental Table 1, were included in the study.
Data exclusions	Participants were excluded if they did not fulfill all of the following eligibility criteria:<br>VA enrollees aged ≥18 years<br>Assigned to VA primary care team in the prior 12 months AND has at least one VA primary care visit in the last 12 months<br>At least one blood pressure measurement in the last 12 months AND weight in the last 5 years AND height ever documented<br>Known residential address and VISN assignment<br>Received at least one documented COVID-19 vaccine in the VA healthcare system at any time in the past<br>Did not receive any COVID-19 vaccination in the prior 3 months<br>Did not receive a Novavax JN.1 COVID-19 vaccine (2024-25 formulation) at any time before<br>Did not receive a KP.2 COVID-19 vaccine at any time before<br>Did not test positive for SARS-CoV-2 in the prior 3 months<br>No inpatient Hospitalization in the prior 30 days
Replication	Data analysis was repeated multiple times during the study. Any inconsistencies in the results were identified and resolved.
Randomization	In this observational study, eight sequential trials were executed each one (except the first and last) consisting of a ~2-week enrollment period beginning on 08/23/24 and continuing until 01/17/25. For each 2-week period, a two-step process was used to match each eligible KP.2 vaccine recipient to an eligible person who did not receive the KP.2 vaccine as of that period, in order to emulate the balance in baseline characteristics achieved by randomization, using exact matching followed by propensity score matching.

Exact-matching
Within each 2-week trial, we first exact-matched each eligible participant who received KP.2 COVID-19 vaccine to all eligible participants who did not receive KP.2 COVID-19 vaccine using six factors:
1. Age Category (18-64, 65-74, ≥75)
2. CAN score 3.0 (90-DAY mortality) category (0-50, 51-89, ≥90)
3. VA Integrated Service Network (VISN), the 18 administrative regions of the VA
4. Time since most recent COVID-19 vaccine (90-182, 183-364 and ≥365 days prior to the beginning of each 2-week trial)
5. Timing of most recent SARS-CoV-2 positive test (90-182, 183-364 and ≥365 days, or no infection documented prior to the beginning of each 2-week trial)
6. Number of primary and specialty outpatient healthcare visits (excluding mental health) in the previous 1 year (0-4, 5-8, 9-15, ≥16)

Propensity-score matching
After exact-matching by these six factors, we performed an additional propensity score matching step ultimately aiming to identify the best-matching comparator. We used matching with replacement in a 1:1 variable ratio with a comparator randomly selected in the case of ties.
The characteristics included in the propensity score logistic regression model are:
1. Demographic:
a. age (continuous),
b. sex,
c. race
d. ethnicity
2. Urban versus rural residence based on RUCA codes
3. Distance to nearest VHA medical facility: <10, 10-24, 25-49, ≥50 miles
4. Substance use (documented in the prior 2 years)
a. tobacco,
b. alcohol use disorder,
c. other substance use disorder
5. Underlying CDC high-risk conditions (documented in the prior 2 years)
a. Body mass index: <18.5, 18.5-24.9, 25-29.9, 30-34.9, 35-39.9, ≥40 kg/m2
b. Chronic kidney disease
c. Diabetes
d. Coronary heart disease
e. Congestive heart failure
f. Chronic lung disease
g. Dementia
6. Charlson Comorbidity Index (continuous)
7. CAN score (continuous)
8. Healthcare utilization: number of primary care encounters in the previous one year, categorized
9. Healthcare utilization: number of specialty care encounters other than mental health in the previous one year, categorized
10. Healthcare utilization: Number of hospitalizations in the last 12 months
11. Receipt of immunosuppressive or cancer medications in the prior 1 year
12. Number of prior COVID-19 vaccinations

Blinding

The analysts were blinded to treatment allocation when extracting the study outcomes.

Reporting for specific materials, systems and methods

We require information from authors about some types of materials, experimental systems and methods used in many studies. Here, indicate whether each material, system or method listed is relevant to your study. If you are not sure if a list item applies to your research, read the appropriate section before selecting a response.

Materials & experimental systems

n/a	Involved in the study
☒	☐ Antibodies
☒	☐ Eukaryotic cell lines
☒	☐ Palaeontology and archaeology
☒	☐ Animals and other organisms
☒	☐ Clinical data
☒	☐ Dual use research of concern
☒	☐ Plants

Methods

n/a	Involved in the study
☒	☐ ChIP-seq
☒	☐ Flow cytometry
☒	☐ MRI-based neuroimaging

Plants

Seed stocks	*Report on the source of all seed stocks or other plant material used. If applicable, state the seed stock centre and catalogue number. If plant specimens were collected from the field, describe the collection location, date and sampling procedures.*
Novel plant genotypes	*Describe the methods by which all novel plant genotypes were produced. This includes those generated by transgenic approaches, gene editing, chemical/radiation-based mutagenesis and hybridization. For transgenic lines, describe the transformation method, the number of independent lines analyzed and the generation upon which experiments were performed. For gene-edited lines, describe the editor used, the endogenous sequence targeted for editing, the targeting guide RNA sequence (if applicable) and how the editor was applied.*
Authentication	*Describe any authentication procedures for each seed stock used or novel genotype generated. Describe any experiments used to assess the effect of a mutation and, where applicable, how potential secondary effects (e.g. second site T-DNA insertions, mosiacism, off-target gene editing) were examined.*
